# A Method of HBase Multi-Conditional Query for Ubiquitous Sensing Applications

**DOI:** 10.3390/s18093064

**Published:** 2018-09-12

**Authors:** Bo Shen, Yi-Chen Liao, Dan Liu, Han-Chieh Chao

**Affiliations:** 1 School of Electronic and Information Engineering, Beijing Jiaotong University, Beijing 100044, China; 14120201@bjtu.edu.cn (Y.-C.L.); 16120093@bjtu.edu.cn (D.L.); 2 Key Laboratory of Communication and Information Systems, Beijing Municipal Commission of Education, Beijing 100031, China; 3 School of Information Science and Engineering, Fujian University of Technology, Fuzhou 350118, China; hcc@niu.edu.tw; 4 School of Mathematics and Computer Science, Wuhan Polytechnic University, Wuhan 430023, China; 5 Department of Electrical Engineering, National Dong Hwa University, Hualien 97401, Taiwan

**Keywords:** ubiquitous sensing, HBase, multi-conditional query, Hilbert space-filling curve

## Abstract

Big data gathered from real systems, such as public infrastructure, healthcare, smart homes, industries, and so on, by sensor networks contain enormous value, and need to be mined deeply, which depends on a data storing and retrieving service. HBase is playing an increasingly important part in the big data environment since it provides a flexible pattern for storing extremely large amounts of unstructured data. Despite the fast-speed reading by RowKey, HBase does not natively support multi-conditional query, which is a common demand and operation in relational databases, especially for data analysis of ubiquitous sensing applications. In this paper, we introduce a method to construct a linear index by employing a Hilbert space-filling curve. As a RowKey generating schema, the proposed method maps multiple index-columns into a one-dimensional encoded sequence, and then constructs a new RowKey. We also provide a *R*-tree-based optimization to reduce the computational cost of encoding query conditions. Without using a secondary index mode, experimental results indicate that the proposed method has better performance in multi-conditional queries.

## 1. Introduction

Nowadays, the trend is getting stronger and stronger that gathered data growth and deployed ubiquitous sensing application systems [1,2,3,4,5] are highly interrelated and mutually improved. The development of ubiquitous sensing, including wireless sensor networks and their applications, speeds up the production of data [6,7,8,9,10]. The value mined from these data further promotes rapid advances in the manufacturing of wireless sensors and the development of the Internet of Things. For example, ubiquitous healthcare applications employ wireless body area networks and any other mobile devices to provide sustainable and reliable collection capability for healthcare data [11,12]. Civil infrastructure monitoring systems, including buildings, bridges, roads and pipeline monitoring applications, collect various state parameters to assess the structural health of target objects synthetically [13,14]. As an extension of ubiquitous sensing, ubiquitous learning applications analyze the learning logs with the aid of seamless learning systems, which gather data using ubiquitous technologies, such as RFID (Radio Frequency Identification), wireless and mobile communications, PDAs, and wearable computers [15,16,17]. All these rely on information retrieval, storage, and data processing [18]. Most data generated by ubiquitous sensing applications have the character of time series, such as monitoring data of power station, and from others a pattern of interrelationship emerges, for instance the correlation between patients, disease, and symptoms. Further, high sampling frequency and high data generation rate also feature. To satisfy the needs of various requirements, a data storage system should have various abilities, such as making different schemes and profiles for different applications.

A traditional relational database can be employed to meet these requirements, but many data tables should be created and many relationships should be maintained, even when only one pattern should be supplied. For example, patients with different diseases should be arranged by their diverse therapeutic regimens. The therapeutic schedule of some patients includes operation, injection, and physiotherapy, while others need to diet or take medicine. When a relational database is used, a patient table, treatment table, and patient–treatment relationship table are needed to record the selection of treatments for each patient. To retrieve the treatment plan of a patient, we first need to query the patient table by patient id or name to get detailed information about the patient, and then query the patient–treatment relationship table by patient id to get all records, and finally query the treatment table to get the treatment name by treatment id. Merging these retrieved data, we will obtain the whole treatment plan of the patient. Of course, a database view may sample this query operation, but the query process is the same. In a ubiquitous sensing environment, data operations like that are universal, especially when the information is from a heterogeneous data source. To meet this demand, the NoSQL database is a better alternative, which employs a key–value structure to support the storage of unstructured data. For the above case, that means each patient has a single record which contains all his treatments. To retrieve the treatment plan, a single query can get all data about a patient. Obviously, the process is simplified.

Time series databases are also developed to store a large number of time series data rapidly, for example, InfluxDB and RRD Tool. It has a similar structural character to a relational database but can only index data in a few columns, which means a measurement has a fixed structure and querying special metrics is not easy. As a kind of simplified structure database, a time series database provides the ability to store more data than a relational database.

HBase, a typical implementation of a distributed NoSQL database, has superior performance in reading and writing when dealing with large-scale data [19,20]. It employs key–value structure as its data storage paradigm to provide support for high-speed querying and scalability. HBase is efficient when querying by RowKey, but also has functional limitations when querying in non-RowKey columns or doing multi-conditional queries [21].

A multi-conditional query uses more than one value as conditions for retrieving qualified data from multiple non-RowKey columns. In a relational database, this kind of query can be effectively executed by building indexes on multiple columns. However, HBase establishes an efficient B+ index for RowKey columns only and does not support auxiliary indexes of other columns natively [22]. This is due to the fact that rows with diverse columns and different numbers of columns are allowed in HBase with an atomic key-value container. To make a multi-conditional query, full table scanning cannot be avoided, which invariably results in terrible performance.

In many application scenarios, searching in more than one column with multiple conditions is common. For example, large e-commerce sites have a large number and a wide variety of goods with many different attributes. Storing these data in a relational database needs several tables, but using HBase, one table is enough because the data of each commodity can be stored in a single row in spite of their different attributes. For instance, storing data about books with title and author attributes in one row of a table and data about clothes with color and style attributes in another row of the same table. This storage mode takes HBase scaling horizontally and also makes it difficult to locate the rows that satisfy the criteria.

One way to solve this problem is to build the secondary index for columns respectively, which uses a similar practice to a relational database and uses the coprocessor mechanism of HBase to maintain consistency of column value and index value. Although the solution improves query performance on non-RowKey columns, it only supports a range search for the first condition when a multi-conditional query executes [23].

Another option is named linearized index [24], which maps multi-dimensional data into one dimension by linearization techniques and then makes use of the native RowKey index of HBase. A linearized index does not need to take up additional storage space or maintain an extra index table but must keep the proximity relationship of points in a multi-dimensional space to enhance the performance of a multi-conditional query. In this kind of solution, how to design the mapping process is the point.

In order to support a multi-conditional query in HBase, we propose a new model to generate a RowKey based on a Hilbert space-filling curve [25]. The model employs the spatial continuity and clustering feature of a Hilbert curve to construct a linearized index for realizing single point query and range search [26,27], which has good load balancing characteristic and is capable of avoiding query hotspots. We then introduce the optimization of the query method based on a multi-dimensional Hilbert index. We also analyze and validate the availability of the proposed method and compare the query performance with other methods. The proposed method provides the ability to query HBase data by any combination of conditions in ubiquitous sensing applications without losing the horizontal scaling function, which makes it more rapid and easy for these applications to get the required data for further special analysis.

## 2. Related Work

HBase shows excellent read-write performance when dealing with big data, but it only supports efficient query by RowKey and does not support indexed multi-conditional queries natively. An efficient query relies on reasonable index structure, so the key to realizing an efficient multi-conditional query is the index design. There are some solutions in the literature, such as specially designed RowKey methods based on secondary indexes and methods based on space-filling curves [28].

The idea of specially designed RowKey attempts to encode query conditions into a RowKey when data is inserted into a database. It utilizes the performance of a RowKey query and prefix filter fully. For example, to quickly retrieve the patients who took a given medicine, such as atorvastatin, the RowKey is likely to have the form of ‘medicine-id:patient-id’. Certainly, hash(medicine-id): hash(patient-id) is another practicable form. Then prefix matching can get the query results easily.

There are several manners of implementing special RowKeys in the literature, including a delimiting mode for variable length value (the first form above for instance), fixed representation mode (the second form above), and single column mode (serialize and store all data into a single column). An alternative way is to put the data value into the column qualifier name, which is indexed in a row.

Obviously, specially designed RowKeys can enhance the query efficiency and support a multi-conditional query when the potential query conditions are encoded into a RowKey in advance. But, on the other hand, it lacks the flexibility to adapt to changing search conditions. Further, data updates need re-encoding of the RowKey, which brings limits the method.

In order to search data effectively by non-RowKey conditions, the secondary index table should be established for those non-RowKey columns. The secondary index maintains a mapping relationship from the non-RowKey column to the RowKey. That is to say the non-RowKey column value of the original table or its hash value becomes the RowKey of the secondary index table and the RowKey of the original table becomes a general column.

After a secondary index is built, a query to a non-RowKey column can be separated into two steps. First, finding a RowKey from the secondary index table by the query condition and then retrieving the final result from the original table by the RowKey that has been found. Although two queries are needed, a secondary index produces a better performance than the full table scan.

Clearly, the heart of a secondary index is how to build and maintain the index when data change. In other words, how to maintain data consistency. HBase just has a function named coprocessor which can be employed to achieve the objective of maintaining consistency. The coprocessor is a native mechanism that can be triggered by inserting new data, deleting old data or updating existing data. The typical implementation of secondary index includes Hindex [29,30] and complementary clustering index (CCI) [31].

Hindex implements the above solution strictly, while CCI makes use of redundant backup data to generate a complementary clustering index table. CCI replaces random searching on the data table with continual scanning on the index table. Usually, only the column with potential query conditions and the RowKey are stored in the secondary index table. When queried, the index table will be searched for RowKeys of the data table first and then the actual results in the data table according to these RowKeys will be found. 

In most cases, the RowKeys obtained from the index table are random, which leads to lots of random reading operations for retrieving data. It is inefficient, especially for a range query. To reduce random queries, CCI stores detailed information of data in the index table. Thus, the data can be found directly by sequential scanning in the index table. Namely, random reading becomes sequential reading, which enhances query performance. Meanwhile, when many columns need to be indexed, the index increases data redundancy. Moreover, it is difficult to update the index when data are modified in the data table. The dynamic creation and update of indexes are not supported in CCI.

Like the secondary index, the method based on a space-filling curve can also introduce a non-invasive implementation of a multi-conditional query to HBase. A space-filling curve is a well-known technique for indexing multi-dimensional data by transforming them into one dimension, such as *Z*-ordering used by MD-HBase [24,32].

There are three steps in MD-HBase index construction. First, data space is divided with a KD-tree. Then the dimensions of the sub-space are reduced and binary crossing codes are generated by *Z*-ordering. Final, the longest common prefix naming is obtained to execute a query. A KD-tree is a kind of binary tree which is used to find the nearest neighbor and approximate nearest neighbor in massive high-dimensional data space. Building a KD-tree on a *k* dimension dataset is just to partition the corresponding *k* dimensional space for mapping each *k* dimensional hyperrectangle to a node on the KD-tree. Due to the continuity of the sub-space and the ergodicity of *Z*-ordering dimensionality reduction in the direction of increasing each dimension, the distribution of *k* dimensional data has not changed after dimensionality reduction by *Z*-ordering.

When a range query is executed, the *Z*-value of the starting condition determines the first sub-space to be scanned and the *Z*-value of ending condition determines the last. The sub-spaces between them are all to be scanned. 

In brief, the basic idea is dividing the space into many small grids and encoding each grid to a code generated by a space-filling curve. Thus, searching for a point in the space can be converted to calculating the code of a grid in which the target point is located. Encoding multi-dimensional data into a single code makes a multi-conditional query possible but space-filling curves may not be friendly to the range query of the float. Furthermore, if the grid is too small, it will lead to many unqualified grids being searched in a query, while if the grid is too large, many unqualified points in the grid will be checked. All these would reduce the search efficiency.

## 3. Multi-Conditional Query Method Based on a Hilbert Space-Filling Curve

Like any other kind of database, the core idea of query efficiency improvement is how to narrow down the search scope, which is usually realized through building an index. The solution based on a linearized index has an advantage in terms of numeric queries. In the past research, *Z*-ordering was widely used for linearization. Some other results indicate that serval curves with good natures can be used, such as a Hilbert curve which is continuous in space and has an excellent clustering feature. Employing a Hilbert curve to construct linear index can realize more efficient multi-conditional single point queries and range queries. It also has good load balancing characteristic which can help avoid the issue of a hot query point [25]. So here we try to use the Hilbert curve to partition a conditional space and implement the structure of a multi-conditional Hilbert value index [33].

### 3.1. Mapping from N-Dimensional to One-Dimensional

**Figure 1:**
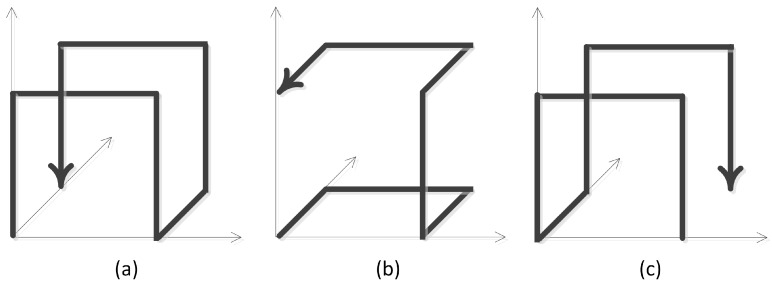
Three-dimensional Hilbert curve unit coordinate transformation. (**a**) Initial state; (**b**) after exchanging; (**c**) after reversing.

Let $R^{N}$ be an *N*-dimensional data space. If a Hilbert curve can fill $2^{m\times N}$ *N*-dimensional hyperrectangular space, the curve is called an *N*-dimensional m-order Hilbert space-filling curve, denoted by $H_{m}^{N}(m\geq 1,N\geq 2)$. $H_{m}^{N}$ can be achieved by $H_{m-1}^{N}$ through coordinate transformation, and $H_{1}^{N}$ is named a Hilbert curve unit of $H_{m}^{N}$, represented by $C^{N}$. The number of $C^{N}$ encoded in consequence in a one-dimensional space is called the Hilbert sequence, denoted as *H*-order. The *N*-dimensional Hilbert gene $G^{N}$ is a coordinate transformation control information list that controls the formation of $H_{m}^{N}$ from $H_{m-1}^{N}$. The coordinate transformation includes exchanging (‘↔’) and reversing (‘′’). For example, Figure 1 shows the three states of the coordinate transformation of a 3-dimensional Hilbert unit, (a) the initial state; (b) the state after exchanging, and (c) the state after reversing.

Here we use a two-dimensional array to describe the Hilbert gene. Let $G^{N}[h][0]$ store the exchanging operation information and $G^{N}[h][1]$ store the inversion operation information, where h∈H-order.

Given $C^{N}$ and $G^{N}$, a complete *N*-dimensional Hilbert space-filling curve can be generated by an iterative process, where $C^{N}$ can be computed from $C^{1}$ by the following equation,

(1) $$C^{1}=\left(\begin{array}{c} 0\\ 1 \end{array}\right),\;C^{N}=\left(\begin{array}{cc} 0 & C^{N-1}\\ 1 & {\left(C^{N-1}\right)}_{N-1}^{\prime } \end{array}\right)\;$$

where ${\left(C^{N-1}\right)}_{N-1}^{\prime }$ represents reversing the value of the *N*-1th dimension of $C^{N-1}$.

To get $G^{N}$, we need to analyze the entrance and exit of $H_{1}^{N}[i]$ first. The entrance information can be obtained by two properties of the curve. (1) $H_{1}^{N}[0]$ and $C^{N}$ have the same entrance point coordinates; (2) the direction of connection for $H_{1}^{N}[i-1]$ and $H_{1}^{N}[i]$ is the same as that for $C^{N}[i-1]$, and $C^{N}[i]$ and the entrance coordinates of $H_{1}^{N}[i]$ must ensure its connection to $H_{1}^{N}[i-1]$.

Exit information can also be acquired by the properties of the curve. (1) if the entrance point of $H_{1}^{N}[i]$ cannot connect to $H_{1}^{N}[i+1]$ directly, the direction from the entrance to the exit of $H_{1}^{N}[i]$ is the same as the connection direction for $C^{N}[i]$ and $C^{N}[i+1]$; (2) in contrast, the direction of $H_{1}^{N}[i]$ should be chosen in the *N* − 1 unchanged dimensions from $C^{N}[i]$ to $C^{N}[i+1]$.

Based on the information of entrance and exit, $G^{N}$ has the following form,

(2) $$\left\{ \begin{array}{l} G^{N}[i][0]=(C^{N}[0]\wedge C^{N}[2^{N}-1])\\ \;\wedge (H_{1}{}^{N}[i][0]\wedge H_{1}{}^{N}[i][1])\\ G^{N}[i][1]=C^{N}[0]\wedge H_{1}{}^{N}[i][0] \end{array}\right.\;$$

where ∧ denotes the bitwise ‘and’.

Given a point P of a *N*-dimensional m order Hilbert space-filling curve, the corresponding Hilbert code *H* can be obtained by Algorithm 1.

**Algorithm 1.** Calculation of H valueInput:v, a point in $R^{N}$
*m*, the order of the Hilbert curveOutput:*H*, the coordinate of v in $R^{1}$, i.e., Hilbert code, *H*-code1:for each order in m2:  Get p which is made from the highest bit binary of each dimension of v.3:  Inquire $C^{N}$ to get the *H*-order corresponding to p
4:  Put *H*-order in the top digit of *H*;5:  if the current order is not the last one6:   Inquire $G^{N}$ to get the coordinate7:   Transformation information of *H*-order8:   Complete the coordinate transformation of the remainder binary code of v
9:   Reset residual value10:  end if11:end for12:return H

### 3.2. Generate RowKey by Hilbert Mapping Values

To implement a multi-conditional query in HBase, the first step is defining the columns to be indexed which determines the dimension *N* of Hilbert space-filling curve, and then the accuracy of columns, i.e., the binary coding bits of the column value, which dictates the order m. After that, $C^{N}$ and $G^{N}$ can be calculated to construct *H*-code. When performing operations of modifying or inserting data, RowKey generation is of two kinds, Hilbert mapping generation and non-index generation.

Hilbert mapping generation is used when inserting or modifying data that contains indexed columns. Taking these column values as inputs of Hilbert mapping to generate the corresponding binary *H*-code and then appending an independent random number *UUID* at the end of the *H*-code to form the RowKey. To ensure the uniqueness of the RowKey, the appended *UUID* is needed. When querying, a filter should be defined to match the special bits of the *H*-code in the RowKey. The RowKey has the following form,

(3) RowKey=H-code:UUID 

Non-index generation is used when the inserted data does not contain an index column. In this case, *UUID* can be used as RowKey directly. Due to the randomness of *UUID*, the probability that the data with this kind of RowKey will be included in the query result is extremely low. Re-filtering can be used to eliminate this data.

### 3.3. Implementation of Query Operation

**Figure 2:**
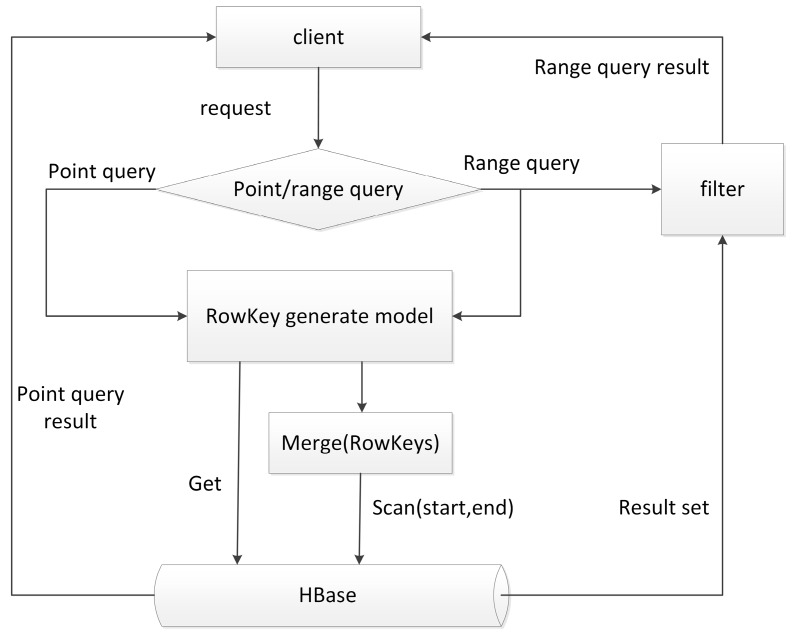
Query process for point query and range query.

The schema proposed above can be implemented in a non-intrusive mode, which means no modification to HBase source code is required. When a point query is requested, the query conditions are first mapped to a *H*-code value, and then a prefix-based query using the HBase API can locate data that meets the conditions. The query process is shown in Figure 2.

If the query is a range query, one or more query conditions is a range, for example, 1000 < column1 < 1011. Relative to the point query, the condition of a range query is a subset of condition space and it is a series of *H*-code segments after being converted to *H*-code. The RowKey corresponding to these *H*-code segments are also some fragments which need to be merged before scanning data. Due to the finiteness of order, consecutive intervals determined by the conditions of the range query may cover a small portion of data which lie outside the query results. Like the case of non-index generation, an additional filter works for removing these data from the query results [34].

### 3.4. Optimization

Although the proposed method for a multi-conditional query can avoid full table scanning, it still brings some additional unavoidable computational cost, such as mapping from multi-condition to *H*-code. Consider that the number of dimensions and order of a Hilbert space curve have been determined during initialization, and that the *H*-code forming RowKey will not change unless the value of the indexed columns is updated, storing the coded query conditions will reduce the computational cost of mapping. When lots of mapping relationships are preserved, how to manage them and quickly find the hotspots of a query should be properly designed. 

Here we use a *R*-tree [35,36] to store these mappings from query conditions to *H*-code, named *H*-code-storing *R*-tree. The basic idea is sorting condition data to be indexed according to the *H*-code of the minimum peripheral matrix center, and then storing these sorted data into leaf nodes by their capacity. After that, we generate an *R*-tree from the bottom up in chronological order.

**Figure 3:**
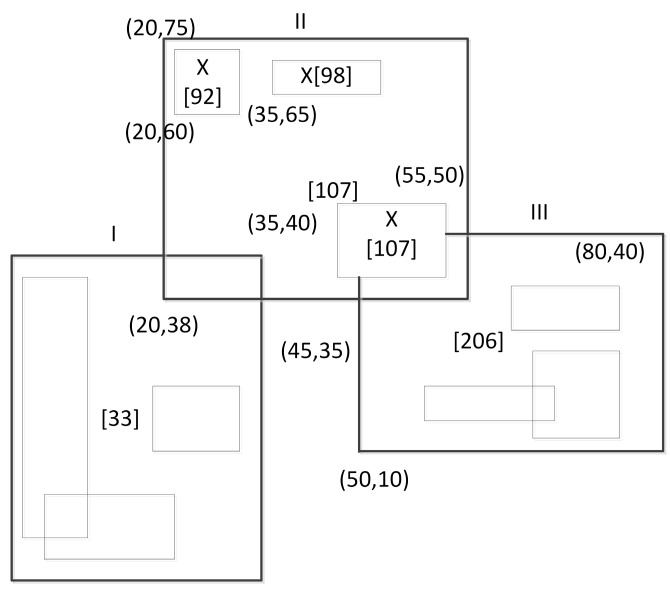
Two-dimensional Hilbert index space division.

The leaf node stores no more than $C_{L}$ pairs (R,H), where R is the smallest enclosing sub-space of data and H is the corresponding *H*-code. Non-leaf nodes store no more than $C_{n}$ triples $\left(R,P_{tr},L\right)$, where $P_{tr}$ is a pointer to a child node, and L is the largest *H*-code value in a sub-space surrounded by *R*. An example of a two-dimensional Hilbert indexing space division is shown in Figure 3. Rectangle I, II, and III are non-leaf nodes and rectangles with X flag are leaf nodes. The numeral in square brackets represents the *H*-code of the node. The query starts from the root node and ends at the leaf node of the tree. All nodes with a query window intersection will be searched, and a *H*-code set that meets requirements will be returned.

## 4. Experimental Results and Discussion

We have implemented the proposed HBase multi-conditional query method based on the Hilbert space-filling curve and its optimization. To evaluate the performance, we deploy a test network with 10 nodes, which include 3 client nodes and a distributed cluster construction of 7 nodes. The cluster has a ZooKeeper node, a HMaster node, and five HRegionServers. We extract the product reviews from the Amazon 2006 Summer Group Buy Network Metadata Collection provided by the Stanford Internet Analysis Project (SNAP) as experimental data, which include 548,552 goods and 7,781,990 reviews. The dataset has features between structural data and un-structural data, which covers the characteristics of data collected by most ubiquitous sensing applications and contains enough data to evaluate the reading and writing performance of algorithms. The structure of each record is as the example shown in Table 1.

**Figure 4:**
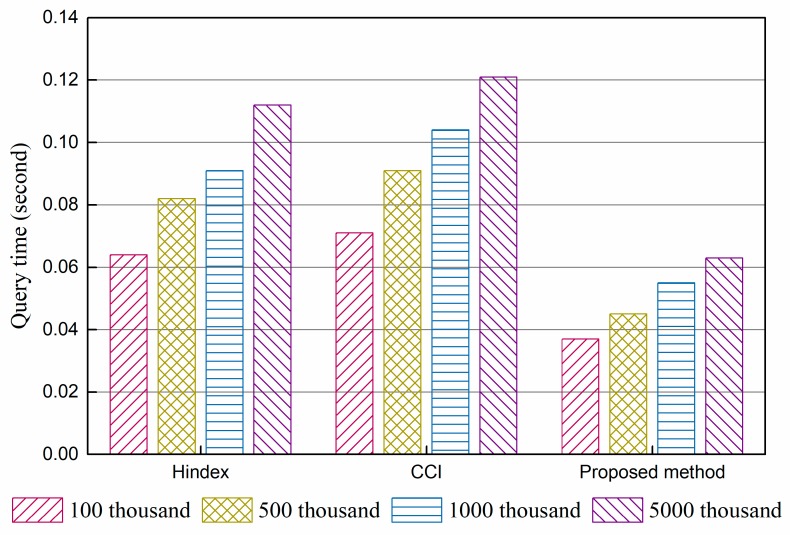
Results of a single-column query performance experiment.

**Figure 5:**
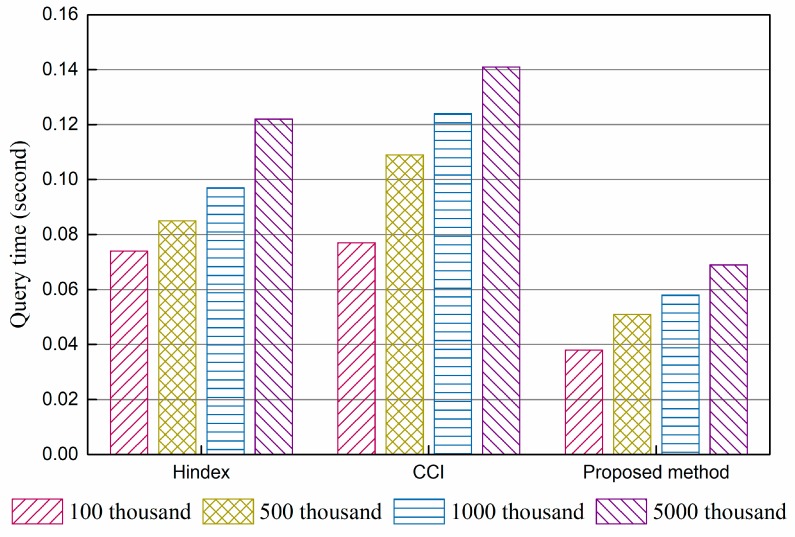
Results of Multi-column query performance experiment.

The query performance of Hindex, complementary clustered indexes (CCI), and the proposed method were compared. The results of the experiments are shown in Figure 4 and Figure 5.

Figure 4 shows the average time per query of a single column consumed by three methods with 100 k, 500 k, 2000 k, and 5000 k data, and Figure 5 gives the results of a multi-column query under the same conditions. The results indicate that there are only some slight differences in query performance between Hindex and CCI under the same query times, while the proposed method takes less time under the condition of both single column query and multi-column query. Hindex uses a secondary index solution, which needs to query the index table before locating data. This two-step query affects the performance in a manner, though all of them are querying on RowKey. CCI employs a complementary clustered index, which writes data into the index table directly and replaces random reading–writing with ordered read–writing. Thus, it has better performance than full table scanning. The performance enhancement of the proposed method benefits from the immediate use of RowKey query, *R*-tree structure, and the removal of a secondary index. From the perspective of the scale of data, the query speed of all three methods slightly declines with the growth in data volumes, because more data regions need to be accessed. The proposed method is the least affected. The results indicate that storing data in regions is beneficial for the expansion capability of HBase but affects query performance unavoidably.

**Figure 6:**
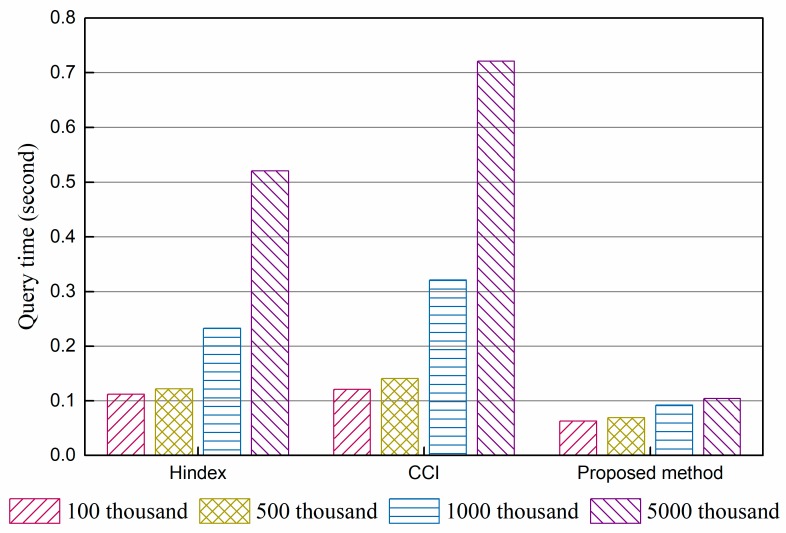
Effect of query conditions.

We also compared the query time of Hindex, CCI, and the proposed method under the condition of one query condition, two query conditions, three query conditions, and four query conditions using 5 million records. The results shown in Figure 6 imply that Hindex has good performance with 1 and 2 query conditions, but the performance begins to decline rapidly when the number of query conditions is more than 3. The detail of implementation indicates that multi-conditional stored in one index table leads to a longer primary key which degrades the query performance [37]. The query performance of CCI also drops sharply with the increase of query conditions [38]. Searching in multiple index tables and combining the resulting data are the reasons. The performance of the proposed method has no marked decline, which is better than the other two schemes. More query conditions need more dimensions of a Hilbert space-filling curve to generate RowKeys, which only leads to some additional computation cost. The increased computations affect the data inserted and updated rather than the query. For most ubiquitous sensing applications, once data are generated and collected into storage systems, they almost do not change. Thus, the proposed method is more suitable for these applications.

**Figure 7:**
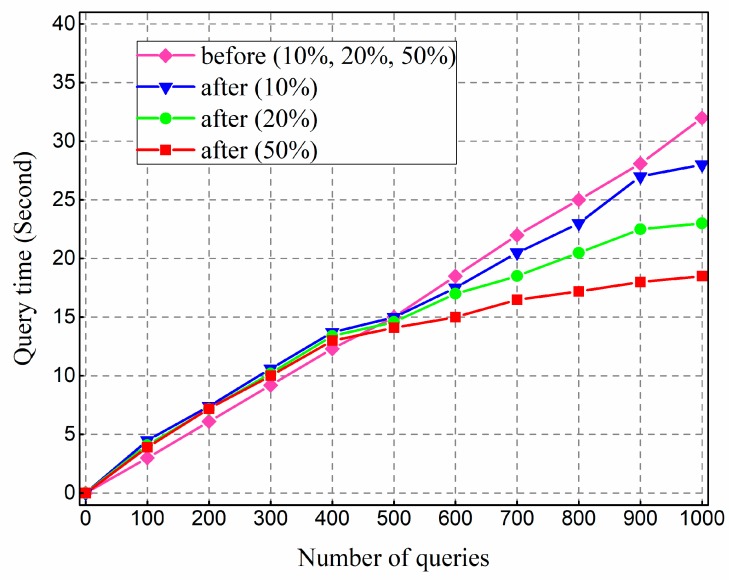
Test results of query optimization.

We also tested the optimization scheme. We built the index on the same columns for the non-optimized system and optimized system respectively, and inserted 2 G data with repetition ratios 10%, 20%, and 50%, and then the query time was counted. We conducted 1000 multi-conditional queries on different repetition ratio data from the three clients respectively and counted the relationship between retrieval time and the number of queries. The results are shown in Figure 7.

The results show that the query performance of the optimized method is slightly inferior to the non-optimized method when the number of queries is small. As the number of query increases, the performance is gradually higher. This trend becomes more significant and rapid as the repetition rate increases. The basic idea of index optimization is to save multiple query conditions into the cache to cut down the repetitive computation cost of *H*-code. *H*-code is calculated at the ZooKeeper node intensively and shared by all clients. Further, when the proportion of hot query data is larger, the query performance can be significantly improved due to direct RowKey querying and cache reading. The results indicate that the cached query condition based on R-tree can effectively improve the performance, which agrees with the design idea of optimization.

## 5. Conclusions

As a principal NoSQL database, HBase provides ubiquitous sensing applications with flexible and convenient data storage abilities, which allows the applications to treat personalized services or differentiated demands as unstructured data directly. On the other hand, HBase still lacks the ability of fast querying by non-RowKey or by multi-condition, which is in popular demand in most application scenarios, particularly in ubiquitous sensing application systems with volumes of data.

For optimizing the query performance of HBase, we proposed a multi-conditional query method, which employs a Hilbert space-filling curve to map multiple conditions into a one-dimensional encoded sequence and then constructs the RowKey of the data table. The proposed method is a non-invasive schema for implementing fast-speed multi-conditional query. To reduce the computational burden of the encoding query condition, we also introduced a method based on R-tree structure to store historical query conditions, which gives a further performance optimization to the query. Experimental results show that the proposed method improves the multi-conditional query performance of HBase effectively. Besides un-structural data, the ability of multi-conditional query makes HBase more suitable for storing structural data and hybrid data, such as time-series data and the description of their meta-data, which are necessary for data analysis in ubiquitous sensing applications. On the other hand, although the proposed method provides better query performance, it still cannot create or modify indexed columns in running time, which means the indexed columns can only be created with table creation and cannot be changed. It will be studied in our future work.

## Figures and Tables

**Table 1:** The structure of data records.

Field Name	Quantity
ASIN	1559362022
Title	Wake Up and Smell the Coffee
Group	Book
Time	13 May 2002
Customer	A2IGOA66Y6O8TQ
Rating	5
Votes	3
Helpful	2
